# Widening Educational Inequalities in Physical Health Due to the Obesity Trend?—A Mediation Analysis Using the German Socio-Economic Panel Study

**DOI:** 10.3389/ijph.2024.1606932

**Published:** 2024-04-29

**Authors:** Stefanie Sperlich, Johannes Beller, Batoul Safieddine, Juliane Tetzlaff, Siegfried Geyer

**Affiliations:** Hannover Medical School, Hanover, Germany

**Keywords:** health inequalites, obesity, time trends, physical health, HRQOL

## Abstract

**Objectives:**

This study examined the contribution of obesity to the development of educational inequalities in physical health.

**Methods:**

We used data from the German Socio-Economic Panel for the period 2002–2020. Physical health was measured with the modified SF12-questionnaire. Logistic regression analyses were applied to estimate time trends. The Relative Index of Inequality (RII) and the Slope Index of Inequality (SII) were calculated to examine educational inequalities. The role of obesity as a mediator was analyzed using the Karlson-Holm-Breen (KHB) method.

**Results:**

Over time, educational inequalities in obesity as well as impaired physical health widened in men and women, particularly among those aged 30–49 years. For individuals with a low level of education at this age, the probability of impaired physical health increased significantly by 7.7%-points in women and 9.4%-points in men. Of this increase, 25.9% for women and 14.8% for men could be attributed to the increase in obesity.

**Conclusion:**

Our findings suggest that the steeper rise in obesity among individuals with a low level of education partly explains the observed widening in educational inequalities in physical health.

## Introduction

In Europe as well as in other western countries, the share of overweight women and men as well as those with obesity has increased, and it is predicted to increase in the years to come [1–3]. According to German national survey-based data from 2019/2020, about 19% of the population were classified as obese [4], making obesity a relevant public health issue. Obesity has effects on the expenditures of the healthcare system, is associated with increased sickness absence, and carries the risk of a multitude of comorbidities [5, 6].

At the individual level, obesity has shown to be associated with depression, with direction of causality running in both directions [7–9]. Moreover, the presence of obesity negatively affects the quality of life, which is partly due to the loss of physical function that accompanies obesity [10]. In a nationwide German study, it was shown that obesity was more prevalent in individuals with lower educational status, and that increasing obesity was associated with increased degrees of impaired physical health. This relationship was stronger in female than in male respondents [11]. Similar results were obtained in an earlier cross-sectional study conducted in the United States in 1998. It included 32,440 respondents and reported evidence for a linear association between physical quality of life and obesity [12]. A cross-sectional survey study from Great Britain examined relationships between obesity and different dimensions of wellbeing measured with the SF-36. It turned out that subjects with both obesity and other chronic conditions reported particularly poor physical and emotional health [13].

A review paper concluded that rising global obesity may be due to the combination of the consumption of high-caloric food, technological innovations that led to reduced physical activity, and changing sociodemographic factors such as such as increased urbanization [14]. The literature emphasizes that obesity should be seen as a social phenomenon where appropriate interventions should target both socio-economic and socio-cultural factors [15, 16]. The importance of social factors is also reflected in the fact that socially disadvantaged people are more affected by obesity [17–19].

Given the association between obesity and physical health, it is reasonable to assume that rising obesity rates have led to rising rates of impaired physical health. This could be particularly true for socially disadvantaged individuals, where obesity rates have increased more sharply over time [17, 18]. For example, Hoebel et al. found that the low and medium socioeconomic groups showed increases in obesity prevalence, whereas no such trend was observed in the high socioeconomic groups [17]. Extensive research has also documented that low socioeconomic status is associated with poor physical health and health functioning [20–22]. Moreover, previous studies suggest that the temporal development of physical and mental health differ according to the age group considered. While it improved in older ages, stable or even worsening trends in younger ages have been reported [23–26]. For trend analyses, this suggests that in addition to differentiation by socio-economic status, differentiation should also be made by age cohort.

To date, few studies have examined the relationship between the temporal trend of obesity and physical health and its impact on the development of health inequalities. This study addressed this research question by first analyzing how rates of obesity and impaired physical health developed in both genders depending on the level of education and two different age cohorts. In a second step, we determine the direction of causality between obesity and physical health using a cross-lagged-panel design. Finally, stratified by educational level, we analyzed the extent to which changes in physical health over time are mediated by changes of obesity rates.

## Methods

### Data Source

The analyses are based on data from the German Socio-Economic Panel study (GSOEP V.31), conducted by the German Institute for Economic Research. The GSOEP is a representative annual survey of German individuals aged 18 and older in private households that started in 1984 [27]. Data were collected by face-to-face interviews using different questionnaires for individuals, households and specific subgroups. The central survey instrument for this study is an individual questionnaire on socio-demographic characteristics and the health-related-quality of life, which each adult household member is supposed to answer. The GSOEP population is updated regularly with new survey samples to reflect changes in the German population and in order to compensate for dropouts occurring over time. Further information on GSOEP can be obtained from Goebel et al. [27].

We included participants between 30 and 64 years of age and performed all analyses stratified for the age groups 30 to 49 and 50–64 years since former studies revealed substantial differences according to the age groups considered. Our analyses are based on a pooled dataset including the waves from 2002 to 2020, allowing for trend analysis on a population level by means of cross-sectional comparisons. We used cross-sectional weights that are designed to produce a nationally representative sample.

Overall, our study included 51,718 respondents (women: 26,417/men: 25,301) and 164,165 observations (women: 87,088/men: 77,077). Respondents with missing information were excluded. The study was conducted according to the STROBE cross sectional reporting guidelines [28].

### Measures

#### Physical Health

Physical health was measured by the physical component summary score (PCS-score) using a slightly modified version of the second version of the 12-Item Short Form Health Survey (SF-12v.2). In the GSOEP, the SF-12v.2 is available since 2002 (wave S) and is provided every second year. Physical health contains six items measuring physical functioning, role limitations due to physical problems, physical pain and general health. Based on these items, the physical PCS-score was calculated. Values are standardized to a national norm (GSOEP population in 2004), ranging from 0 to 100 points with a mean of 50 points and a standard deviation of 10 points [29]. A higher score corresponds to a better health status. We used a dichotomous variable with norm values (t-values) < 40 points indicating a significant deviation of more than a standard deviation below population average. We used this variable, which indicates significant impairment in physical health, to show not only statistical but also clinically significant changes over time, which is not as possible when looking at changes in mean values [30].

#### School Education

All individuals with a maximum of 9 years of schooling were assigned to the low educational group that includes also subjects without a school leaving certificate. The intermediate education group consists of those with 10 years of schooling corresponding to a comprehensive school certificate. Subjects with at least 12 years’ schooling were assigned to the high educational group, which corresponds to the university entrance qualification.

#### Time Trend

A categorical variable covering five time-periods (from 2002/04 to 2018–2020) was used to analyze the time trends, with the first period being set to the reference category. We used a 4-year interval to smooth out the outlier years and to compensate for the small sample size in some subgroups, which was particularly the case for impaired physical health in the 30–49 age group. For analyzing temporal change in educational inequalities and the mediation analysis, we used a continuous time trend variable, coded 0 for 2002 and 1 for 2020, with the years in between getting fractional values, for example, 0.1 for 2004, 0.2 for 2006 and so forth. The value obtained from this variable indicates the average change in relation to the respective outcome over the entire period.

#### Obesity

According to the WHO classification, a BMI of 30 or higher was defined as obesity [31]. Weight and height were asked during the face-to-face interview. BMI was calculated as weight in kilograms divided by the height in meters squared. In 11.1% of the sample, information on weight or height was not requested, so that no BMI could be calculated. The proportion of missing values is 1.2%.

### Statistical Analyses

We analyzed the temporal development of obesity (BMI ≥30) and impaired physical health (PCS < t-value 40), stratified by gender, age groups, and levels of school education by means of logistic regression analyses, adjusted for age and nationality. The categorical time trend variable as the independent variable includes five time-points with the first time-period used as the reference category. We used cluster-robust standard errors to adjust for the panel structure of the data. In addition to odds ratios (OR), we estimated predicted probabilities to visualize the results. For determining the predicted probabilities, we used the interaction term “time trend*level of school education.” This was done to avoid possible bias due to different age distributions in the educational groups, which may be present in stratified analyses.

For analyzing trends in educational inequalities in obesity and impaired physical health, we calculated the Relative Index of Inequality (RII) and the Slope Index of Inequality (SII) [32]. RII represents the prevalence ratio between subjects with the lowest and the highest educational level while the SII quantifies the magnitude of absolute health inequality between individuals at the top and bottom of the educational hierarchy. In order to calculate RII and SII, the educational groups of each time period (4-years interval) and for both age groups (separated for men and women) were transformed into cumulative rank probabilities (“ridit scores”). As proposed, we used a logarithmic link function to calculate the RII and an identity link function to calculate the SII by using clustered variance estimators [32]. Based on RII and SII, temporal trends in educational inequalities in obesity and impaired physical health were assessed by the inclusion of a two-way interaction term between the ridit scores and the continuous time trend variable. The first point in time of the persons with the highest level of education represents the reference category. Accordingly, interaction-terms with RII >1 and SII >0 indicate increasing health inequalities to the disadvantage of individuals with the lowest level of education.

Moreover, we performed a cross-lagged panel analysis in order to describe the directional influences between body mass index (BMI) and physical health (PCS), using the “sem” command by STATA that fits structural equation models [33]. As an example, we have analyzed the causal relationship between BMI and PCS with a time interval of 4 years for the most recent survey periods. This means that we compared the relationship between BMI in 2016 and PCS in 2020 with the relationship between PCS in 2016 and BMI in 2020. As part of sensitivity analyses, we conducted further analyses with time intervals other than 4 years, i.e. 6, 8, and 10 years.

Finally, based on logistic regression analysis, we applied the Karlson-Holm-Breen-method (KHB-method) to examine how much of the time-effect on impaired physical health is mediated by temporal changes of obesity-rates. The KHB approach extends the decomposition properties of linear models to logistic regression models by decomposing the total time-effect on physical health into a direct and indirect effect. This method ensures that the crude and adjusted coefficients are unaffected by the rescaling bias that arise in cross-model comparisons of non-linear models. In our case, the *total effect* in terms of Odds Ratio (OR) is the effect of time on impaired physical health without the mediator, only controlled for age, nationality and the residual variance. The *direct effect* of time corresponds to the effect that is left after controlling for obesity as a potential mediator. Accordingly, the *indirect effect* is the share of the time-effect on impaired physical health that is explained by the temporal change in obesity. The indirect effect in terms of OR is calculated as the total effect divided by the direct effect. In addition to ORs, we reported average partial effects (APE) giving the decomposition a more substantial interpretation. APE are measured on the probability scale and estimate the average marginal effect of the mediator as expressed in %-points [34]. With respect to APE, the indirect effect is calculated by the total effect minus the direct effect.

All regression analyses were performed separately for men and women and stratified by age groups and level of education. We controlled for age and nationality (German versus other), taking possible shifts in age composition and the number of non-German residents into account. Population weights were employed to match the official population statistics. All analyses were performed with STATA v13.1 [35].

## Results

The weighted sample characteristics stratified by obesity-status are displayed in Table 1. The proportion of missing values on the variables included varied between 0% and 1.3%. It shows that compared to women, men were more frequently obese (53.7% vs. 46.3%). Moreover, obese individuals were more often socially disadvantaged in terms of school education, occupational position, employment status and adjusted household net income (Table 1).

**TABLE 1:** Weighted sample characteristics in % by obesity-status (German Socio-Economic Panel study, 2002–2020).

	BMI <30 *n* = 116,246	BMI ≥30 *n* = 27,659
Gender		
men	50.0	53.7
women	50.0	46.3
missing^a^	0	0
Educational level		
low	28.7	38.8
intermediate	32.1	32.3
high	29.6	19.4
others	9.7	9.5
missing	1.3	1.0
Occupational position^b^		
low	17.3	22.7
intermediate	53.4	55.0
high	29.3	22.3
missing	0.3	0.3
Employment status		
full-time	55.0	51.4
part-time	28.1	25.1
not employed	17.0	23.4
missing	0	0
Unemployed^c^		
yes	6.7	9.3
no	93.1	90.7
missing	0.3	0.2
Household net income^d^		
<60% median	9.5	14.4
60% - < 150%	65.3	66.9
≥150%	25.3	18.7
missing	1.6	2.0
Nationality		
German	89.1	88.7
others	10.9	11.3
missing	0	0

Notes: numbers of observations.

^a^ The missing values are not added together with the valid values to 100%, but are added separately.

^b^ Occupational position: low: unskilled, semi-skilled and skilled workers, farmers, salaried employees with simple activities and civil servants in the ordinary service, intermediate: self-employed persons without employees, salaried employees with qualified activities and civil servants in the middle civil service, high: self-employed persons with employees, salaried employees with highly qualified jobs, master/mistress, civil servants in the upper and higher levels of the civil service.

^c^ Unemployed means searching for a job.

^d^ Based on modified equivalence scale: <60% of the median household net income (poverty risk threshold), between 60% and 150% of the median household net income and >150% of the median household net income, *n* = maximum number of observations.

### Temporal Development of Obesity by Age Group and School Education

**FIGURE 1:**
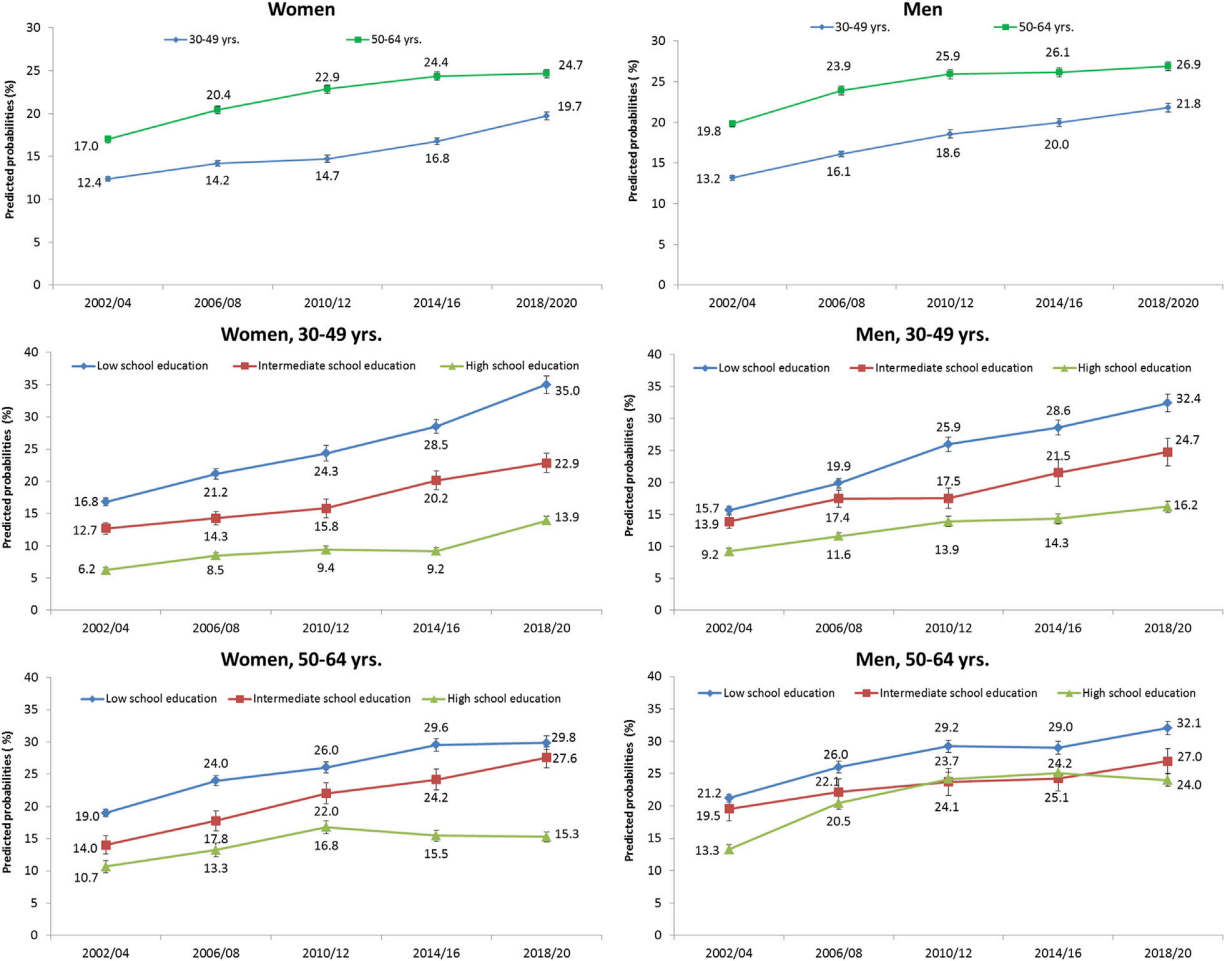
Predicted probabilities and standard errors (SE) of obesity (BMI ≥30) from 2002/04 to 2018/20 in men and women, stratified by age group and school education (German Socio-Economic Panel study, 2002–2020).

From 2002/04 to 2018/20, the predicted probabilities of obesity (BMI ≥30) increased in women and men, irrespective of age and educational level (Figure 1). For both genders, individuals aged 30–49 years with a low level of education had the highest absolute increase with a rise from 16.8% to 35.0% in women and from 15.7% to 32.4% in men. In relative terms, the odds of obesity in this age group more than doubled in 2018/20 compared with the baseline (2002/04) for both genders and for all levels of education (Supplementary Table S1).

In terms of RII and SII, we found significant educational inequalities in obesity for each time point considered (Table 2, upper part). As the interaction term obesity*trend indicate, the magnitude of difference between the highest and lowest educational level (SII) increased over time for individuals aged 30 to 49, with a steeper rise in women (SII: 0.20, CI 0.14–0.26) as compared to men (SII: 0.11, CI 0.05–0.17). Compared to individuals aged 30 to 49, the inequalities were less pronounced among 50 to 64-year-olds. In contrast to the SII, the obesity ratio between people with the lowest and highest level of education (RII) remained largely stable for both genders and age groups.

**TABLE 2:** Relative and absolute inequalities in obesity (BMI ≥30) and impaired physical health (PCS < t-value 40), Relative Index of Inequality (RII) and Slope Index of Inequality (SII), stratified by time, gender and two age groups (German Socio-Economic Panel study, 2002–2020).

Age-group	Time	Obesity (BMI ≥30)							
		Women				Men			
		RII	95% CI	SII	95% CI	RII	95% CI	SII	95% CI
30–49 years	2002/04	4.11***	3.09; 5.48	0.16***	0.13; 0.19	2.20***	1.69; 2.85	0.11***	0.08; 0.14
	2006/08	3.84***	2.99; 5.09	0.18***	0.14; 0.22	2.50***	1.94; 3.24	0.15***	0.11; 0.19
	2010/12	5.10***	3.83; 6.82	0.23***	0.19; 0.27	2.60***	2.01; 3.37	0.17***	0.13; 0.22
	2014/16	5.38***	4.32; 6.70	0.29***	0.26; 0.33	2.69***	2.12; 3.42	0.19***	0.14; 0.23
	2018/20	4.57***	3.69; 5.66	0.32***	0.28; 0.36	2.80***	2.23; 3.53	0.22***	0.17; 0.26
	Obesity*Trend	*1.26*	*0.85; 1.86*	0.20***	*0.14; 0.26*	*1.22*	*0.83; 1.79*	0.11**	*0.05; 0.17*
50–64 years	2002/04	2.81***	2.05; 3.87	0.16***	0.11; 0.21	2.04***	1.57: 2.67	0.14***	0.09; 0.19
	2006/08	2.72***	2.05; 3.62	0.19***	0.13; 0.24	1.73***	1.35; 2.22	0.13***	0.07; 0.19
	2010/12	2.08***	1.63; 2.66	0.16***	0.11; 0.21	1.60***	1.28; 2.00	0.12***	0.06; 0.17
	2014/16	2.48***	1.78; 3.11	0.20***	0.15; 0.26	1.51***	1.21; 1.88	0.11***	0.05; 0.16
	2018/20	2.92***	2.39; 3.58	0.26***	0.21; 0.30	1.84***	1.51; 2.25	0.16***	0.11; 0.21
	Obesity*Trend	*1.01*	*0.69; 1.49*	0.09*	*0.01; 0.17*	*0.90*	*0.64; 1.27*	*0.00*	*−0.08; 0.09*

		Impaired physical health (PCS < t-value 40)							
		Women				Men			
Age-group	Time	RII	95% CI	SII	95% CI	RII	95% CI	SII	95% CI
30–49 years	2002/04	2.54***	1.87; 3.46	0.07***	0.05; 0.09	3.61***	2.55; 5.11	0.07***	0.05; 0.09
	2006/08	3.01***	2.09; 4.33	0.08***	0.05; 0.10	4.19***	2.90; 6.07	0.09***	0.07; 0.12
	2010/12	2.89***	2.02; 4.14	0.09***	0.06; 0.12	6.15***	4.29; 8.82	0.13***	0.10; 0.16
	2014/16	5.78***	4.46; 7.50	0.18***	0.16; 0.21	7.14***	5.21; 9.78	0.15***	0.13; 0.18
	2018/20	5.71***	4.29; 7.62	0.18***	0.15; 0.21	13.85***	9.24; 20.77	0.18***	0.15; 0.20
	PCS < 40*Trend	2.79***	*1.80; 4.31*	0.14***	*0.10; 0.17*	4.04***	*2.51; 6.52*	0.11***	*0.08; 0.15*
50–64 years	2002/04	1.96***	1.55; 2.47	0.16***	0.11; 0.21	3.78***	2.92; 4.89	0.27***	0.22; 0.32
	2006/08	2.18***	1.73; 2.75	0.18***	0.13; 0.23	3.73***	2.85; 4.88	0.26***	0.21; 0.31
	2010/12	2.25***	1.81; 2.81	0.18***	0.13; 0.23	3.04***	2.40; 3.84	0.24***	0.18; 0.27
	2014/16	2.74***	2.21; 3.42	0.23***	0.18; 0.27	4.33***	3.42; 5.49	0.29***	0.25; 0.34
	2018/20	3.59***	2.96; 4.34	0.28***	0.24; 0.32	5.10***	4.10; 6.35	0.31***	0.27; 0.35
	PCS < 40*Trend	1.96***	*1.43; 2.67*	0.13***	*0.06; 0.20*	1.43*	*1.01; 2.04*	*0.05*	*−0.01; 0.12*

Notes: RII: Relative Index of Inequality (prevalence ratio of the highest and lowest educational level), SII: Slope Index of inequality (magnitude of difference between the highest and lowest educational level); *p < 0.05, **p < 0.01, ***p < 0.001.

### Temporal Development of Physical Health by Age Group and School Education

**FIGURE 2:**
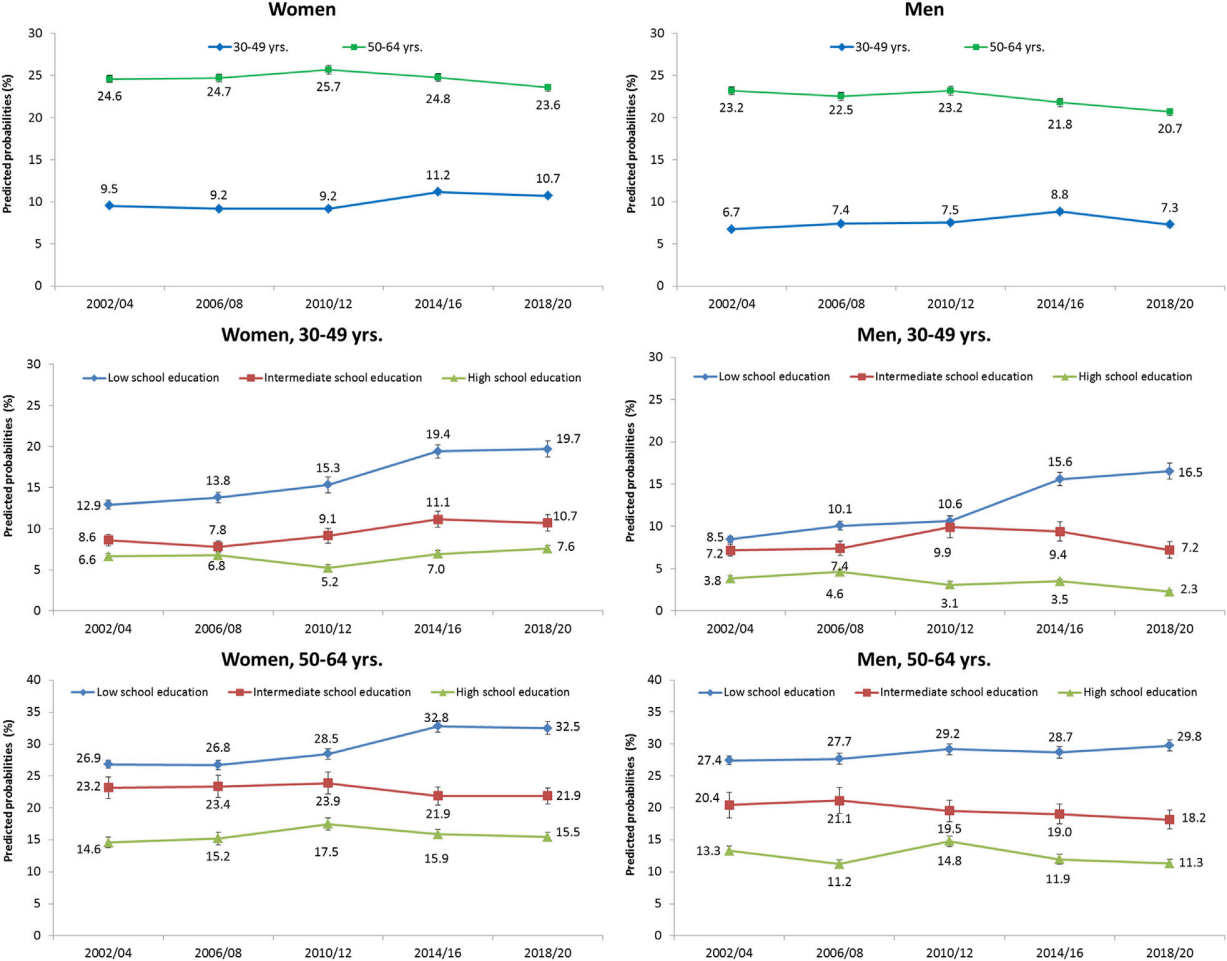
Predicted probabilities and standard errors (SE) of impaired physical health (PCS < t-value 40) from 2002/04 to 2018/20 in men and women, stratified by age group and school education (German Socio-Economic Panel study, 2002–2020).

Among women and men aged 30–49 years, predicted probabilities of impaired physical health (PCS < t-value 40) slightly increased over time from 9.5% to 10.7% and 6.7%–7.3%, respectively (Figure 2). As the corresponding odds ratios indicate, physical health has not changed substantially over time (Supplementary Table S2). Women and men aged 50–64 years had a moderate decrease in the predicted probabilities of impaired physical health from 24.6% to 23.6% and 23.2%–20.7%, respectively. A significant increase in physical health impairment was found from 2014/16 onwards for educationally disadvantaged women and men aged 30 to 49 and also for educationally disadvantaged women aged 50 to 64 (Supplementary Table S2).

Educational inequalities in physical health were found to be significant for both genders at each time point considered (Table 2, lower part). As the interaction terms PCS<40*Trend show, health inequalities for women and men increased over time, both for RII and SII, with the exception of SII for men aged 50–64 years. Again, the increase was more pronounced among people aged 30 to 49 than among people aged 50 to 64.

### Temporal Development of Impaired Physical Health by Obesity Status

Stratified by obesity status, it turned out that women and men with obesity showed considerably higher probabilities of impaired physical health compared to their non-obese counterparts, and this holds for each time period considered. These obesity-related differences in the extent of impaired physical health tended to widen over time, in particular among women aged 30–49 years (Supplementary Figure S1).

### Direction of Causality Between Obesity and Limited Physical Health

In order to determine the direction of causality between BMI and physical health (PCS), we performed a cross-lagged panel analysis (Supplementary Figure S2). It turned out that the relationship between BMI in 2016 and PCS in 2020 was significant while the relationship between PCS in 2016 and BMI in 2020 was not. This finding suggests that BMI acts on PCS rather than PCS on BMI. The negative signs of the coefficients indicate that PCS declined with increasing BMI.

### Decomposition of the Time-Trend on Impaired Physical Health

**FIGURE 3:**
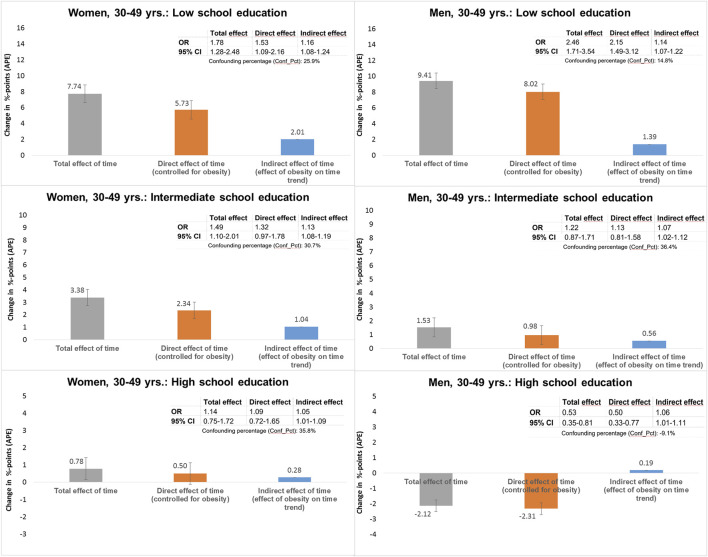
Decomposition of the total time effect on impaired physical health (PCS < t-value 40) into direct and indirect effects via obesity (BMI ≥30) in women and men aged 30–49 years, stratified by school education (German Socio-Economic Panel study, 2002–2020).

In line with Figure 2, the odds of impaired physical health among individuals with a low level of education aged 30–49 years increased significantly from 2002 to 2020, both for women (OR: 1.78, 95%CI: 1.28–2.48) and for men (OR: 2.46, 95%CI: 1.71–3.54) (“total effect,” Figure 3). After controlling for obesity (“direct effect”), this increase was reduced in women and men to OR: 1.53 (95%CI: 1.09–2.16) and OR: 2.15 (95%CI: 1.49–3.12), respectively. A significant effect of time on impaired physical health was found in both genders, which is explained by obesity (“indirect time effect”). Expressed in average partial effects (APEs), the probability of impaired physical health increased significantly by 7.7%-points and 9.4%-points from 2002 to 2020 for women and men with a low level of education, respectively (total time effect). After controlling for obesity, this increase was reduced to 5.7%-points in women and 8.0%-points in men (direct time effect). Accordingly, the contribution of obesity to this increase is 2.0%-points and 1.4%-points, respectively (indirect time effect). This means that for women and men with a low level of education, 25.9% and 14.8% of the increase can be attributed to the increase in obesity, respectively (Conf_Pct., Figure 3).

A significant effect of time was also found for women with intermediate school education. In this group, 1.0%-points of the total increase in the probability of impaired health of 3.4%-points could be attributed to the increase in obesity, which corresponds to a proportion of 30.7% (Conf_Pct) of the total time effect. For women with a high level of education and men with an intermediate level of education, only small increases in the probability of impaired physical health were found. However, a significant effect of obesity is also observed here, explaining 35.8% and 36.4% of the increase in women and men, respectively. Men aged 30–49 years with a high level of education showed the opposite trend of a decrease in the probability of impaired physical health, which are slightly more pronounced when controlled for obesity. As indicated by the Conf_Pct = −9.1%, the decrease would have been 9.1% greater (APE: −2.3%-points instead of −2.1%-points) if there had been no increase in obesity.

**FIGURE 4:**
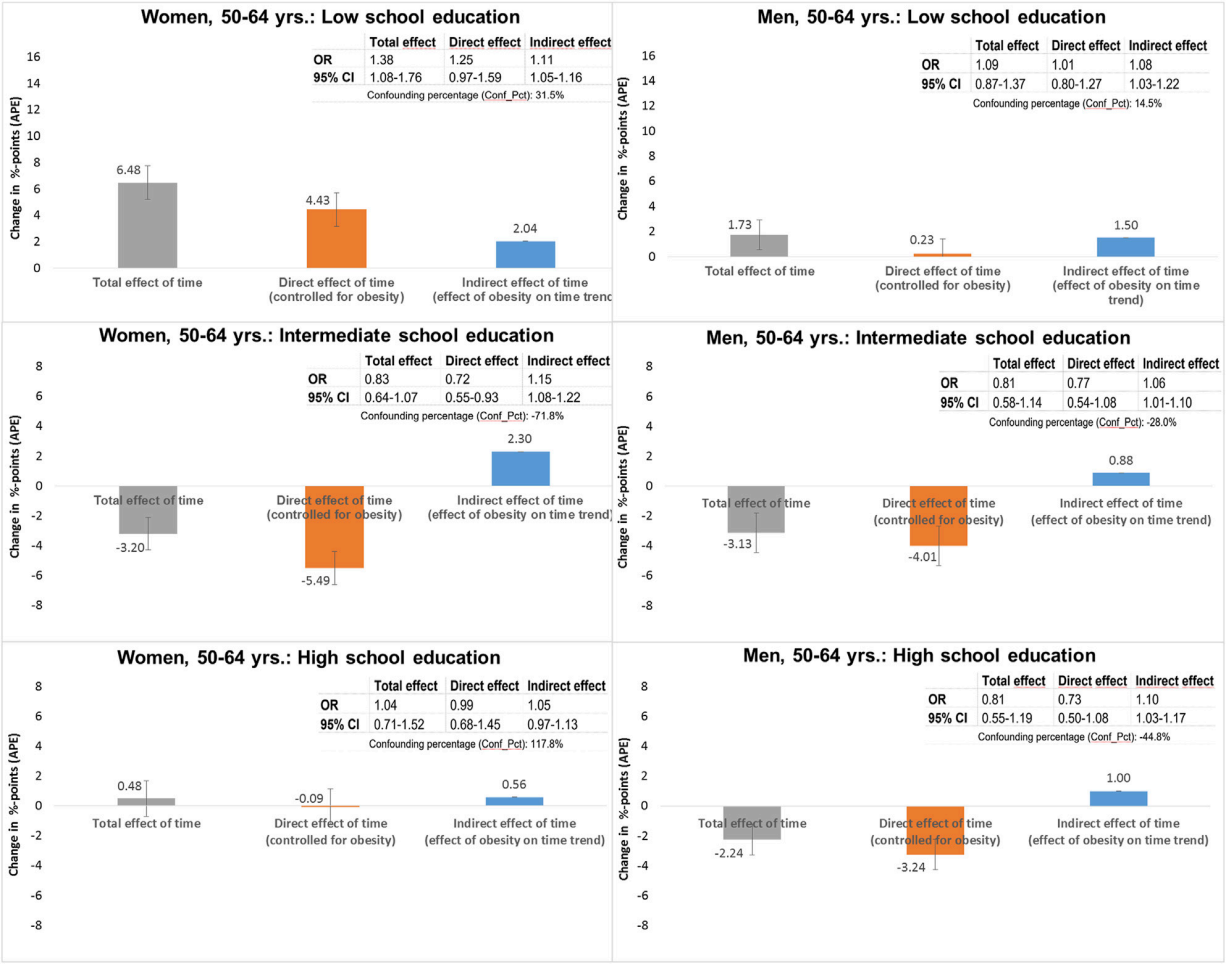
Decomposition of the total time effect on impaired physical health (PCS < t-value 40) into direct and indirect effects via obesity (BMI ≥30) in women and men aged 50–64 years, stratified by school education (German Socio-Economic Panel study, 2002–2020).

Figure 4 shows the corresponding findings for the age group 50–64 years. At this age, a significant increase in the probability of impaired physical health was found among women with a low level of education. Here, 2.0%-points of the total 6.5%-point increase could be attributed to the effect of obesity, corresponding to 31.5% of the total effect of time. A significant effect of obesity was also found in individuals with an intermediate level of education and in men with a high level of education, for whom the probability of impaired physical health decreased over time. For example, the decrease in the probability of impaired physical health among women with intermediate level of education would have been 2.3%-points higher (APE: −5.5%-points instead of −3.2%-points) if there had been no increase in obesity.

## Discussion

As a main finding of our analyses, it turned out that obesity rates were rising over time in women and in men over all age groups considered, and rates were increasing more strongly with decrasing educational levels. As a consequence, we found widening educational inequalities in obesity, in particular with respect to the magnitude of difference between the highest and lowest educational level (SII). This unfavourable development is in line with international figures where rates were increasing over time at the population level. In the United States, the Center for Disease Control reported an overall obesity rate of 42.4% in 2017/2018 [36]. In the United Kingdom, 26% of the general population was affected in 2021, and in all high-income countries, the figures are on the rise with social inequalities to the detriment of people in lower socio-economic positions being present [37]. The most likely reason for this development is the increased consumption of high-caloric food, easy accessibility of fast food supply and an increasingly sedentary lifestyle [38–40]. However, the exclusive consideration of behavioral causes of obesity falls short, as behavior is largely the result of environmental and social conditions that strongly influence personal choices [16]. Our finding that obesity in Germany is increasing more strongly among socially disadvantaged people emphasizes the importance of preventive measures that target social conditions in tackling obesity. Studies on network analyses have shown that obesity spreads along social networks [41, 42]. A setting approach in which relevant influencers of the social network are involved in supporting health behavior change could therefore be an effective strategy.

### Direction of Association Between Obesity and Impaired Physical Health

In line with previous findings [11] we found that obese individuals showed considerably higher rates of impaired physical health compared to their non-obese counterparts, holding for each time period considered. Testing the direction of association, we found that the body weight measured with the BMI apparently acts on physical health rather than physical health on BMI. This finding supports our theoretical assumption that rising obesity rates lead to an increase in physical impairments. However, it should be noted that the reverse can also be true. This means that health impairments can lead to a lack of exercise, which can increase the BMI.

### The Influence of the Obesity Trend on the Development of Educational Inequalities in Physical Health

In contrast to the continuous rise in obesity, the development of physical health was inconsistent. However, in individuals with a low level of education aged 30–49 years, we found a marked increase in rates of impaired physical health that corresponded to the strong increase in obesity rates in this group. Moreover, a significant increase in the proportion of impaired physical health was also found among low educated women aged 50–64 years. In contrast, women and men with higher levels of education showed only a slight increase or even a decrease in the proportions of impaired physical health, which has led to increasing educational inequality in physical health in both absolute and relative terms.

Using the decomposition analysis technique, we found that among men and women aged 30–49 years with low educational attainment, 14.8% and 25.9% of the 7.7 %-point and 9.4%-point increase in physical health impairment, respectively, was due to the increase in obesity. For women aged 50–64 years with the same level of education, the corresponding figures were a share of 31.5% on the 6.5%-point increase in impaired physical health. Obesity was also found to have an effect in individuals with a medium and a high level of education, where predominantly a decline in the rates of impaired physical health was observed. It revealed that this decline would have been greater if there had been no increase in obesity. Our findings are consistent with a previous study from the United States that found that increasing BMI partially explains the trend toward increasing disability and functional impairment among adults [43].

Overall, we found that obesity could partly, but not fully, explain the increase in impaired physical health in people with a low level of education. The question arises as to what other factors could have contributed to the widening of health inequalities. The study by Havet and Penot suggested that inequalities in work exposure increased between 2003 and 2017, particularly to the detriment of blue-collar workers. For example, the situation of shift workers deteriorated in terms of the exposure to vibrations and awkward postures [44]. Taking up this possible explanatory approach, further studies should investigate how working conditions have changed over time for different educational and occupational groups and how this could be related to the development of physical health over time.

### Limitations

We used respondent-based measures of physical health as outcome. Although such measures are important health indicators, they do not inform about specific diagnoses and may thus have some limitations. In order to be substantiated empirically, diagnoses need to be assessed and examined along with subjectively perceived health. Moreover, in our study, the BMI may be underestimated because weight and height were self-reported and people may tend to report a lower weight and higher height due to social desirability. For the cross-lagged panel model, it should be noted that the consideration of several points in time increases the significance of the findings. We therefore calculated additional analyses with time intervals other than 4 years, i.e. 6, 8, and 10 years between t1 and t2. These analyses confirmed the described causal relationship between BMI and physical health. However, the cross-lagged panel cannot solve the problem of confounding and self-selection, which must be taken into account when interpreting the findings. In addition, it might be possible that the time-trends in impaired physical health are biased by the exclusion of the institutionalized population and persons who could not participate in the survey for health reasons [45]. The panel structure of the data could lead to selective attrition, as participants’ health could deteriorate over time and this could lead to dropout. However, there is no reason to assume that this affected the reported time trends as long as the health bias did not increase over time. A shift in the perception of physical health may also have contributed to changes in the proportions of impaired physical health over time. The changes observed may therefore also be due to changes in norms and values regarding physical health. Finally, it should be noted that we have used the classical mediation approach as introduced by Baron and Kenny [46]. In future analyses, the application of causal mediation analysis based on the counterfactual framework [47] would be a useful extension to confirm the present findings.

### Conclusion

Our findings suggest that educational inequalities in obesity as well as in physical limitations widened between 2002 and 2020, particularly among individuals aged 30–49 years. The steeper rise in obesity among those with a low level of education contributed in part to the observed widening in educational inequalities in physical health. Among individuals with a higher level of education, we also found an effect of obesity, predominantly in the way that the positive trend of a decrease in the rate of impaired physical health would have been stronger if there had been no increase in obesity. Further studies are needed to investigate which other factors in the respective educational groups have contributed to the negative or positive temporal development of physical health.

## Supplementary Material

SUPPLEMENTARY FIGURE S1 Predicted probabilities and standard errors (SE) of impaired physical health (PCS < t-value 40) between 2002/04 and 2018/20 in men and women, stratified by obesity status, German Socio-Economic Panel study, 2002–2020.

SUPPLEMENTARY FIGURE S2 Causal pathways between body mass index (BMI) and physical health (PCS) between 2016 and 2020 based on cross-lagged panel model, German Socio-Economic Panel study, 2002–2020, ε1/ ε2 = error variance.

## References

[1] OECD, Union E. Health at a Glance: Europe 2022. State of Health in the EU Cyle. Paris: OECD Publishing (2022). 10.1787/507433b0-en

[2] OECD. The Heavy Burden of Obesity 2019. The Economics of Prevention. OECD Health Policy Studies. Paris: OECD Publishing (2019). 10.1787/67450d67-en

[3] WHO. WHO European Regional Obesity Report 2022. Kopenhagen. Regional Office for Europe: World Health Organization (2022).

[4] Schienkiewitz A Kuhnert R Blume M Mensink GBM. Übergewicht und Adipositas bei Erwachsenen in Deutschland - Ergebnisse der Studie GEDA 2019/2020-EHIS. J Health Monit (2022)(3) 23–31. 10.25646/10292

[5] Schienkiewitz A Mensink GB Scheidt-Nave C. Comorbidity of Overweight and Obesity in a Nationally Representative Sample of German Adults Aged 18-79 Years. BMC Public Health (2012) 12:658. 10.1186/1471-2458-12-658 22894173 PMC3526457

[6] Heo M Pietrobelli A Wang D Heymsfield SB Faith MS. Obesity and Functional Impairment: Influence of Comorbidity, Joint Pain, and Mental Health. Obesity (Silver Spring) (2010) 18(10):2030–8. 10.1038/oby.2009.400 19893503

[7] Luppino FS de Wit LM Bouvy PF Stijnen T Cuijpers P Penninx BW Overweight, Obesity, and Depression: A Systematic Review and Meta-Analysis of Longitudinal Studies. Arch Gen Psychiatry (2010) 67(3):220–9. 10.1001/archgenpsychiatry.2010.2 20194822

[8] Jantaratnotai N Mosikanon K Lee Y McIntyre RS. The Interface of Depression and Obesity. Obes Res Clin Pract (2017) 11(1):1–10. 10.1016/j.orcp.2016.07.003 27498907

[9] Mannan M Mamun A Doi S Clavarino A. Prospective Associations between Depression and Obesity for Adolescent Males and Females- A Systematic Review and Meta-Analysis of Longitudinal Studies. PLoS One (2016) 11(6):e0157240. 10.1371/journal.pone.0157240 27285386 PMC4902254

[10] Roderka MN Puri S Batsis JA. Addressing Obesity to Promote Healthy Aging. Clin Geriatr Med (2020) 36(4):631–43. 10.1016/j.cger.2020.06.006 33010899 PMC7533351

[11] Truthmann J Mensink GBM Bosy-Westphal A Hapke U Scheidt-Nave C Schienkiewitz A. Physical Health-Related Quality of Life in Relation to Metabolic Health and Obesity Among Men and Women in Germany. Health Qual Life Outcomes (2017) 15(1):122. 10.1186/s12955-017-0688-7 28601090 PMC5466792

[12] Livingston EH Ko CY. Use of the Health and Activities Limitation index as a Measure of Quality of Life in Obesity. Obes Res (2002) 10(8):824–32. 10.1038/oby.2002.111 12181392

[13] Doll HA Petersen SE Stewart-Brown SL. Obesity and Physical and Emotional Well-Being: Associations between Body Mass index, Chronic Illness, and the Physical and Mental Components of the SF-36 Questionnaire. Obes Res (2000) 8(2):160–70. 10.1038/oby.2000.17 10757202

[14] Bleich SN Cutler D Murray C Adams A. Why Is the Developed World Obese? Annu Rev Public Health (2008) 29(1):273–95. 10.1146/annurev.publhealth.29.020907.090954 18173389

[15] McLaren L. Socioeconomic Status and Obesity. Epidemiologic Rev (2007) 29(1):29–48. 10.1093/epirev/mxm001 17478442

[16] WHO. WHO Acceleration Plan to Stop Obesity (2023).

[17] Hoebel J Kuntz B Kroll LE Schienkiewitz A Finger JD Lange C Socioeconomic Inequalities in the Rise of Adult Obesity: A Time-Trend Analysis of National Examination Data from Germany, 1990-2011. Obes Facts (2019) 12(3):344–56. 10.1159/000499718 31167203 PMC6696774

[18] Meng F Nie P Sousa-Poza A. Obesity Inequality and Well-Being in Germany. Econ Hum Biol (2023) 49:101236. 10.1016/j.ehb.2023.101236 36867949

[19] Anekwe CV Jarrell AR Townsend MJ Gaudier GI Hiserodt JM Stanford FC. Socioeconomics of Obesity. Curr Obes Rep (2020) 9(3):272–9. 10.1007/s13679-020-00398-7 32627133 PMC7484407

[20] Hemingway H Nicholson A Stafford M Roberts R Marmot M. The Impact of Socioeconomic Status on Health Functioning as Assessed by the SF-36 Questionnaire: The Whitehall II Study. Am J Public Health (1997) 87(9):1484–90. 10.2105/ajph.87.9.1484 9314801 PMC1380974

[21] Kivimäki M Batty GD Pentti J Shipley MJ Sipilä PN Nyberg ST Association between Socioeconomic Status and the Development of Mental and Physical Health Conditions in Adulthood: A Multi-Cohort Study. Lancet Public Health (2020) 5(3):e140–9. 10.1016/S2468-2667(19)30248-8 32007134

[22] Sperlich S Klar M-K Safieddine B Tetzlaff F Tetzlaff J Geyer S. Life Stage-Specific Trends in Educational Inequalities in Health-Related Quality of Life and Self-Rated Health between 2002 and 2016 in Germany: Findings from the German Socio-Economic Panel Study (GSOEP). BMJ Open (2021) 11(3):e042017. 10.1136/bmjopen-2020-042017 PMC7934728 33664070

[23] Sperlich S Tetzlaff J Geyer S. Trends in Good Self-Rated Health in Germany between 1995 and 2014: Do Age and Gender Matter? Int J Public Health (2019) 64(6):921–33. 10.1007/s00038-019-01235-y 30918976

[24] Wolff JK Nowossadeck S Spuling SM. Altern nachfolgende Kohorten gesünder? Selbstberichtete Erkrankungen und funktionale Gesundheit im Kohortenvergleich. In: Mahne K Wolff JK Simonson J Tesch-Römer C, editors. Altern im Wandel: Zwei Jahrzehnte Deutscher Alterssurvey (DEAS). Wiesbaden: Springer Fachmedien Wiesbaden (2017). p. 125–38.

[25] Clause-Verdreau AC Audureau É Leplège A Coste J. Contrasted Trends in Health-Related Quality of Life across Gender, Age Categories and Work Status in France, 1995-2016: Repeated Population-Based Cross-Sectional Surveys Using the SF-36. J Epidemiol Community Health (2019) 73(1):65–72. 10.1136/jech-2018-210941 30301764

[26] Greaney ML Cohen SA Blissmer BJ Earp JE Xu F. Age-Specific Trends in Health-Related Quality of Life Among US Adults: Findings from National Health and Nutrition Examination Survey, 2001-2016. Qual Life Res (2019) 28(12):3249–57. 10.1007/s11136-019-02280-z 31482430

[27] Goebel J Grabka MM Liebig S Kroh M Richter D Schröder C The German Socio-Economic Panel (SOEP). Jahrbücher für Nationalökonomie und Statistik (2019) 239(2):345–60. 10.1515/jbnst-2018-0022

[28] Skrivankova VW Richmond RC Woolf BAR Yarmolinsky J Davies NM Swanson SA Strengthening the Reporting of Observational Studies in Epidemiology Using Mendelian Randomization: The STROBE-MR Statement. Jama (2021) 326(16):1614–21. 10.1001/jama.2021.18236 34698778

[29] Nübling M Andersen HH Mühlbacher A Schupp J Wagner G. Computation of Standard Values for Physical and Mental Health Scale Scores Using the SOEP Version of SF12v2. J. of Contextual Economics – Schmollers Jahrbuch (2007) 127(1):171–82. 10.3790/schm.127.1.171

[30] Ranganathan P Pramesh CS Buyse M. Common Pitfalls in Statistical Analysis: Clinical versus Statistical Significance. Perspect Clin Res (2015) 6(3):169–70. 10.4103/2229-3485.159943 26229754 PMC4504060

[31] WHO. Obesity and Overweight (2024). Available from: https://www.who.int/news-room/fact-sheets/detail/obesity-and-overweight (Accessed April 23, 2024).

[32] Mackenbach JP Kunst AE. Measuring the Magnitude of Socio-Economic Inequalities in Health: An Overview of Available Measures Illustrated with Two Examples from Europe. Soc Sci Med (1997) 44(6):757–71. 10.1016/s0277-9536(96)00073-1 9080560

[33] Kearney MW. Cross-Lagged Panel Analysis. In: Allen MR, editor. Sage Encyclopedia of Communication Research Methods. Thousand Oaks: Sage (2017).

[34] Kohler U Karlson K Holm A. Comparing Coefficients of Nested Nonlinear Probability Models. Stata J (2011) 11(3):420–38. 10.1177/1536867x1101100306

[35] Stata_Corp. Stata Statistical Software: Release 14. College Station, TX: Stata Corp (2016).

[36] CDC CfDC. US Obesity Rates Have Tripled over the Last 60 Years (2023). Available from: https://usafacts.org/articles/obesity-rate-nearly-triples-united-states-over-last-50-years/ (Accessed April 23, 2024).

[37] OECD. Health at a Glance: Europe 2020: State of Health in the EU Cycle. Paris: OECD Publishing (2020). p. 237.

[38] Temple NJ. The Origins of the Obesity Epidemic in the USA-Lessons for Today. Nutrients (2022) 14(20):4253. 10.3390/nu14204253 36296935 PMC9611578

[39] Gearhardt AN Schulte EM. Is Food Addictive? A Review of the Science. Annu Rev Nutr (2021) 41(1):387–410. 10.1146/annurev-nutr-110420-111710 34152831

[40] Beller J Graßhoff J Safieddine B. Differential Trends in Prolonged Sitting Time in Europe: A Multilevel Analysis of European Eurobarometer Data from 2013 to 2022. J Public Health (2023). 10.1007/s10389-023-02090-1

[41] Christakis NA Fowler JH. The Spread of Obesity in a Large Social Network over 32 Years. New Engl J Med (2007) 357(4):370–9. 10.1056/NEJMsa066082 17652652

[42] Christakis NA Fowler JH. Friendship and Natural Selection. Proc Natl Acad Sci U S A. (2014) 111:10796–801. 10.1073/pnas.1400825111 25024208 PMC4113922

[43] Zajacova A Huzurbazar S Todd M. Gender and the Structure of Self-Rated Health across the Adult Life Span. Soc Sci Med (2017) 187:58–66. 10.1016/j.socscimed.2017.06.019 28654822 PMC5554534

[44] Havet N Penot A. Trends in Exposures to Physically Demanding Working Conditions in France in 2003, 2010 and 2017. Eur J Public Health (2022) 32(1):73–9. 10.1093/eurpub/ckab195 34788439 PMC9090172

[45] Beller J Geyer S Epping J. Health and Study Dropout: Health Aspects Differentially Predict Attrition. BMC Med Res Methodol (2022) 22(1):31. 10.1186/s12874-022-01508-w 35094681 PMC8802529

[46] Baron RM Kenny DA. The Moderator-Mediator Variable Distinction in Social Psychological Research: Conceptual, Strategic, and Statistical Considerations. J Pers Soc Psychol (1986) 51(6):1173–82. 10.1037//0022-3514.51.6.1173 3806354

[47] Valente MJ Rijnhart JJM Smyth HL Muniz FB MacKinnon DP. Causal Mediation Programs in R, Mplus, SAS, SPSS, and Stata. Struct Equ Modeling (2020) 27(6):975–84. 10.1080/10705511.2020.1777133 33536726 PMC7853644

