# Supplementary figures and images for: Widening Educational Inequalities in Physical Health Due to the Obesity Trend?—A Mediation Analysis Using the German Socio-Economic Panel Study
Source: Int J Public Health. 2024 Apr 29;69:1606932. doi: 10.3389/ijph.2024.1606932 (PMC11089185; doi:10.3389/ijph.2024.1606932)

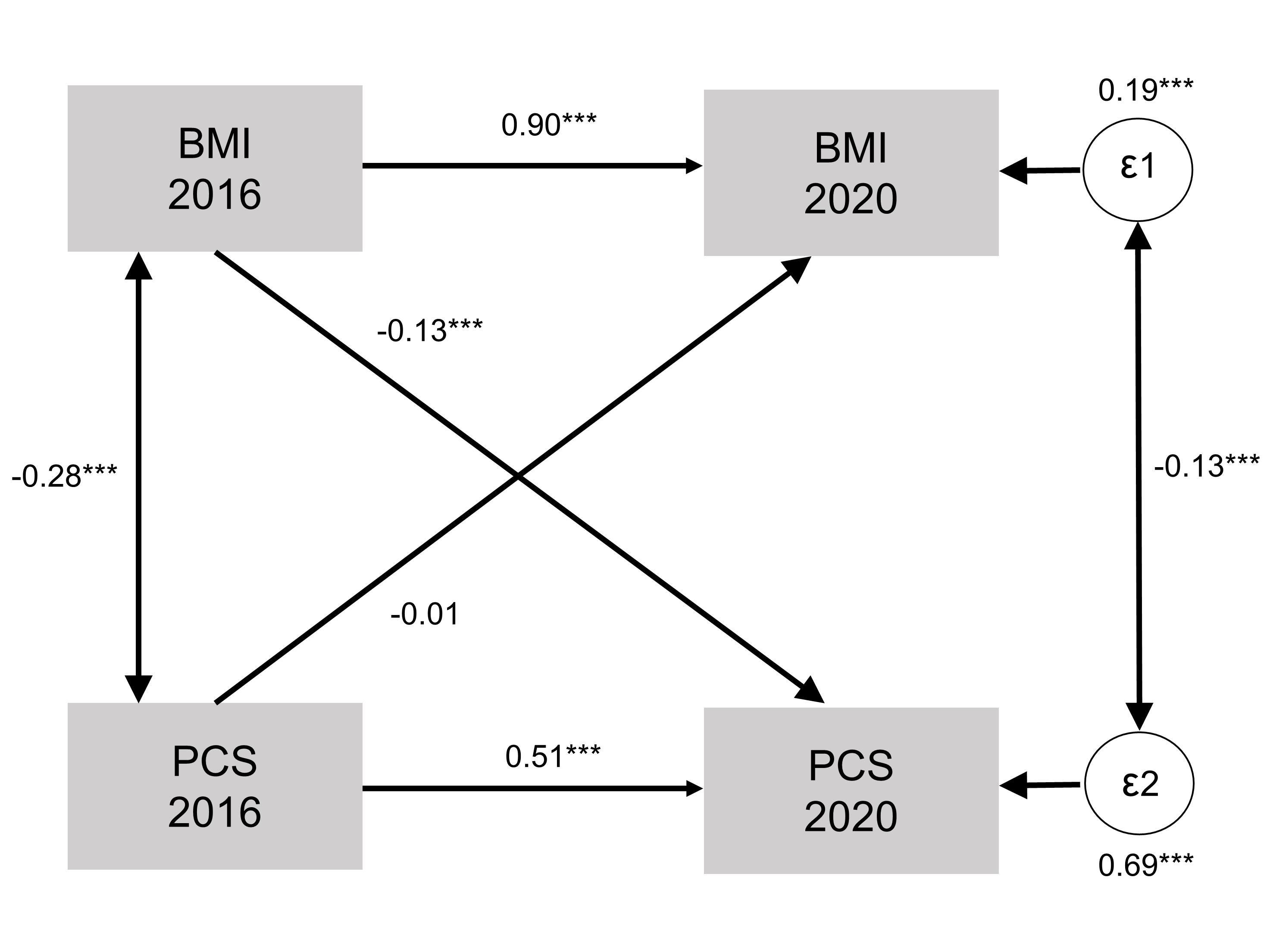

Supplement: Supplementary file 1 [file Image2.TIF]

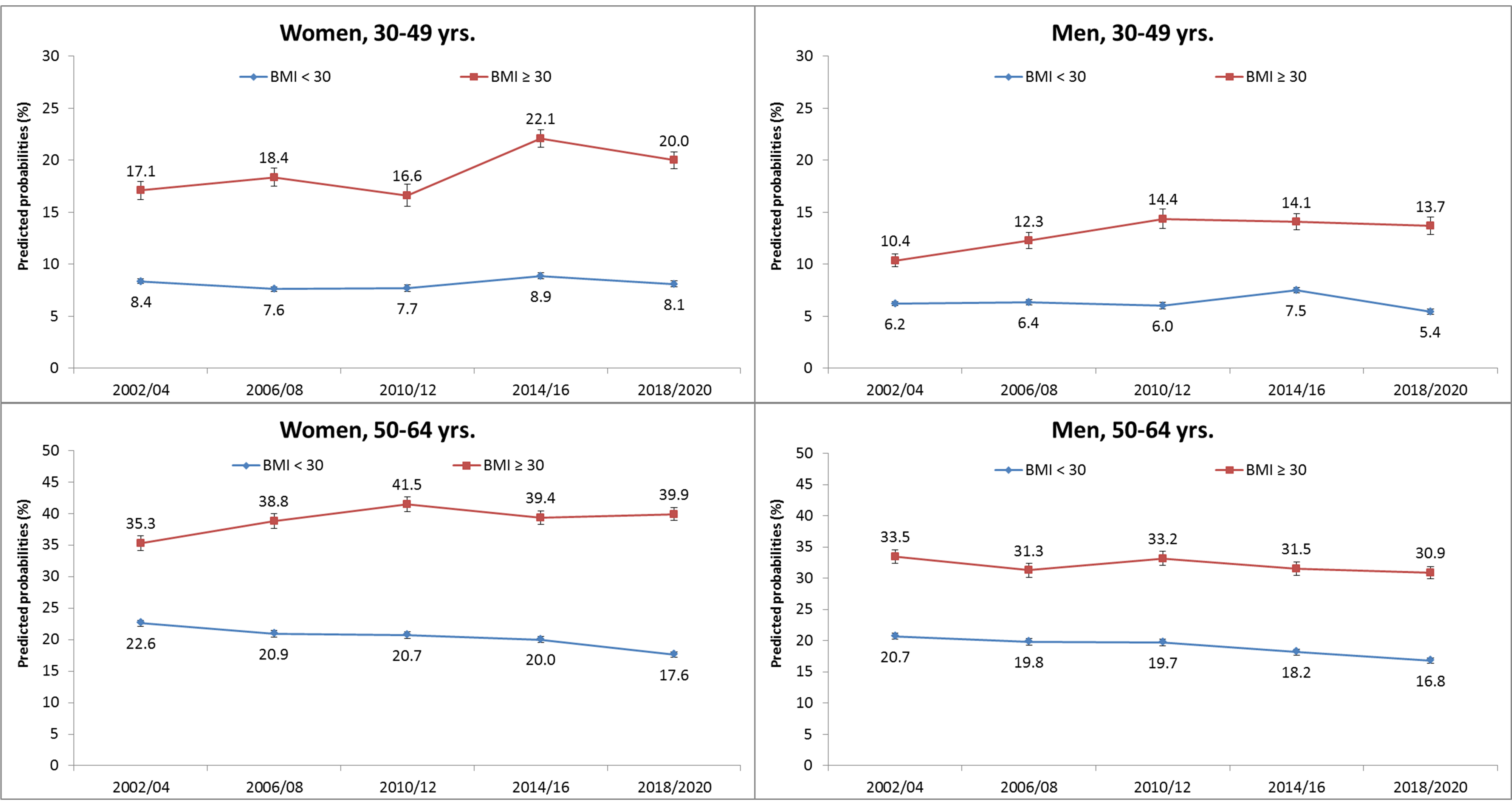

Supplement: Supplementary file 2 [file Image1.TIF]
