# Supplementary material for: Widening Educational Inequalities in Physical Health Due to the Obesity Trend?—A Mediation Analysis Using the German Socio-Economic Panel Study
Source: Int J Public Health. 2024 Apr 29;69:1606932. doi: 10.3389/ijph.2024.1606932 (PMC11089185; doi:10.3389/ijph.2024.1606932)
Supplement: Supplementary file 3 [file DataSheet1.PDF]

Appendix Table S1: Temporal development of obesity (BMI ≥ 30) between 2002/04 and 2018/20 in women and men, stratified by two age groups and school education, German Socio-Economic Panel study, 2002-2020

| | **Total** | | | **Stratified by school education** | | | | | | | | |
|---|---|---|---|---|---|---|---|---|---|---|---|---|
| | | | | **low** | | | **intermediate** | | | **high** | | |
| | **OR** | **95% CI** | | **OR** | **95% CI** | | **OR** | **95% CI** | | **OR** | **95% CI** | |
| **Women:** | | **lower** | **upper** | | **lower** | **upper** | | **lower** | **upper** | | **lower** | **upper** |
| **30-49 yrs.** | | | | | | | | | | | | |
| 2002/04 | 1 | | | 1 | | | 1 | | | 1 | | |
| 2006/08 | 1.17** | 1.06 | 1.30 | 1.33** | 1.10 | 1.60 | 1.15 | 0.98 | 1.33 | 1.40* | 1.08 | 1.81 |
| 2010/12 | 1.22** | 1.05 | 1.42 | 1.61** | 1.22 | 2.11 | 1.29* | 1.02 | 1.64 | 1.56* | 1.10 | 2.21 |
| 2014/16 | 1.42*** | 1.24 | 1.64 | 1.96*** | 1.52 | 2.53 | 1.74*** | 1.38 | 2.19 | 1.52* | 1.07 | 2.16 |
| 2018/20 | 1.74*** | 1.50 | 2.01 | 2.64*** | 2.00 | 3.49 | 2.04*** | 1.61 | 2.58 | 2.44*** | 1.72 | 3.45 |
| **50-64 yrs.** | | | | | | | | | | | | |
| 2002/04 | 1 | | | 1 | | | 1 | | | 1 | | |
| 2006/08 | 1.26*** | 1.12 | 1.41 | 1.37*** | 1.17 | 1.60 | 1.33** | 1.08 | 1.64 | 1.29 | 0.88 | 1.88 |
| 2010/12 | 1.45*** | 1.26 | 1.66 | 1.52*** | 1.24 | 1.86 | 1.73*** | 1.34 | 2.25 | 1.71** | 1.15 | 2.53 |
| 2014/16 | 1.58*** | 1.36 | 1.82 | 1.80*** | 1.43 | 2.26 | 1.94*** | 1.47 | 2.56 | 1.53 | 0.98 | 2.38 |
| 2018/20 | 1.60*** | 1.38 | 1.86 | 1.87*** | 1.46 | 2.38 | 2.34*** | 1.77 | 3.10 | 1.51 | 0.97 | 2.37 |
| **Men** | | | | | | | | | | | | |
| **30-49 yrs** | | | | | | | | | | | | |
| 2002/04 | 1 | | | 1 | | | 1 | | | 1 | | |
| 2006/08 | 1.27*** | 1.15 | 1.39 | 1.33*** | 1.15 | 1.54 | 1.33** | 1.12 | 1.57 | 1.28* | 1.01 | 1.61 |
| 2010/12 | 1.50*** | 1.30 | 1.74 | 1.88*** | 1.47 | 2.42 | 1.34* | 1.07 | 1.68 | 1.57** | 1.14 | 2.17 |
| 2014/16 | 1.65*** | 1.42 | 1.91 | 2.14*** | 1.64 | 2.79 | 1.72*** | 1.29 | 2.30 | 1.63** | 1.19 | 2.23 |
| 2018/20 | 1.84*** | 1.57 | 2.15 | 2.55*** | 1.92 | 3.40 | 2.04*** | 1.54 | 2.72 | 1.90*** | 1.38 | 2.63 |
| **50-64 yrs.** | | | | | | | | | | | | |
| 2002/04 | 1 | | | 1 | | | 1 | | | 1 | | |
| 2006/08 | 1.27*** | 1.14 | 1.42 | 1.30** | 1.11 | 1.53 | 1.18 | 0.94 | 1.48 | 1.68*** | 1.29 | 2.18 |
| 2010/12 | 1.41*** | 1.24 | 1.62 | 1.54*** | 1.26 | 1.87 | 1.30 | 0.98 | 1.73 | 2.08*** | 1.52 | 2.84 |
| 2014/16 | 1.43*** | 1.23 | 1.65 | 1.52*** | 1.21 | 1.90 | 1.34 | 0.99 | 1.79 | 2.19*** | 1.59 | 3.03 |
| 2018/20 | 1.49*** | 1.28 | 1.72 | 1.71*** | 1.36 | 2.16 | 1.53** | 1.14 | 2.06 | 2.07*** | 1.50 | 2.85 |

Notes: Logistic regression of obesity (BMI ≥ 30) on time (reference category: 2002/04), adjusted for age. OR = odds ratios, 95%CI = 95% confidence interval, *p<0.05, **p<0.01, ***p<0.001

Appendix Table S2: Temporal development of impaired physical health (PCS < t-value 40) between 2002/04 and 2018/20 in men and women, stratified by two age groups and school education, German Socio-Economic Panel study, 2002-2020

| | **Total** | | | **Stratified by school education** | | | | | | | | |
|---|---|---|---|---|---|---|---|---|---|---|---|---|
| | | | | **low** | | | **intermediate** | | | **high** | | |
| | **OR** | **95% CI** | | **OR** | **95% CI** | | **OR** | **95% CI** | | **OR** | **95% CI** | |
| **Women** | | **lower** | **upper** | | **lower** | **upper** | | **lower** | **upper** | | **lower** | **upper** |
| **30-49 yrs.** | | | | | | | | | | | | |
| 2002/04 | 1 | | | 1 | | | 1 | | | 1 | | |
| 2006/08 | 0.96 | 0.84 | 1.10 | 1.08 | 0.87 | 1.34 | 0.90 | 0.72 | 1.13 | 1.02 | 0.74 | 1.41 |
| 2010/12 | 0.96 | 0.81 | 1.14 | 1.23 | 0.90 | 1.68 | 1.08 | 0.82 | 1.42 | 0.77 | 0.53 | 1.13 |
| 2014/16 | 1.19* | 1.02 | 1.39 | 1.62*** | 1.24 | 2.11 | 1.34* | 1.03 | 1.74 | 1.04 | 0.73 | 1.50 |
| 2018/20 | 1.14 | 0.97 | 1.34 | 1.64** | 1.21 | 2.22 | 1.28 | 0.97 | 1.68 | 1.15 | 0.81 | 1.64 |
| **50-64 yrs.** | | | | | | | | | | | | |
| 2002/04 | 1 | | | 1 | | | 1 | | | 1 | | |
| 2006/08 | 1.01 | 0.90 | 1.13 | 1.00 | 0.85 | 1.17 | 1.01 | 0.82 | 1.25 | 1.05 | 0.72 | 1.52 |
| 2010/12 | 1.06 | 0.93 | 1.21 | 1.09 | 0.90 | 1.32 | 1.04 | 0.82 | 1.32 | 1.24 | 0.85 | 1.80 |
| 2014/16 | 1.01 | 0.89 | 1.15 | 1.33** | 1.08 | 1.63 | 0.93 | 0.73 | 1.18 | 1.10 | 0.78 | 1.55 |
| 2018/20 | 0.94 | 0.83 | 1.08 | 1.32* | 1.06 | 1.64 | 0.93 | 0.73 | 1.18 | 1.07 | 0.76 | 1.51 |
| **Men** | | | | | | | | | | | | |
| **30-49 yrs**. | | | | | | | | | | | | |
| 2002/04 | 1 | | | 1 | | | 1 | | | 1 | | |
| 2006/08 | 1.11 | 0.95 | 1.29 | 1.21 | 0.96 | 1.52 | 1.04 | 0.78 | 1.37 | 1.21 | 0.83 | 1.77 |
| 2010/12 | 1.13 | 0.93 | 1.38 | 1.28 | 0.96 | 1.70 | 1.43* | 1.02 | 2.00 | 0.80 | 0.45 | 1.43 |
| 2014/16 | 1.34** | 1.13 | 1.60 | 1.99*** | 1.47 | 2.67 | 1.34 | 0.98 | 1.84 | 0.92 | 0.62 | 1.37 |
| 2018/20 | 1.09 | 0.89 | 1.33 | 2.13*** | 1.53 | 2.95 | 1.00 | 0.71 | 1.42 | 0.59*** | 0.38 | 0.91 |
| **50-64 yrs.** | | | | | | | | | | | | |
| 2002/04 | 1 | | | 1 | | | 1 | | | 1 | | |
| 2006/08 | 0.96 | 0.85 | 1.09 | 1.01 | 0.85 | 1.19 | 1.04 | 0.81 | 1.34 | 0.84 | 0.63 | 1.14 |
| 2010/12 | 1.00 | 0.87 | 1.15 | 1.08 | 0.89 | 1.32 | 0.94 | 0.70 | 1.26 | 1.15 | 0.81 | 1.64 |
| 2014/16 | 0.92 | 0.80 | 1.07 | 1.06 | 0.86 | 1.31 | 0.91 | 0.67 | 1.24 | 0.91 | 0.62 | 1.33 |
| 2018/20 | 0.86* | 0.75 | 0.99 | 1.12 | 0.91 | 1.38 | 0.86 | 0.64 | 1.17 | 0.84 | 0.59 | 1.20 |

Notes: Logistic regression of impaired physical health (PCS < t-value 40) on time (reference category: 2002/04), adjusted for age. OR = odds ratios, 95%CI = 95% confidence interval
